# Exercise Improves Alzheimer’s Disease Phenotype in the TgF344-AD Rat, a Behavioral Time Course Study of Males and Females

**DOI:** 10.3390/brainsci15060631

**Published:** 2025-06-12

**Authors:** Stephanie E. Hall, Zachary J. White, Troy T. Rohn, Keshari H. Sudasinghe, Michael E. Young

**Affiliations:** 1 Department of Anatomy and Physiology, College of Veterinary Medicine, Kansas State University, 1600 Denison Ave., Manhattan, KS 66506, USAkeshari93@vet.k-state.edu (K.H.S.); 2 Department of Biological Sciences, College of Arts and Sciences, Boise State University, Boise, ID 83725, USA; trohn@boisestate.edu; 3 Department of Psychological Sciences, College of Arts and Sciences, Kansas State University, Manhattan, KS 66506, USA; michaelyoung@ksu.edu

**Keywords:** Alzheimer’s disease, exercise, TgF344-AD, memory, motor coordination, muscular strength

## Abstract

Alzheimer’s disease (AD) is the third leading cause of death among older adults with nearly 6 million diagnosed annually. In the race for a cure, one thing is certain—exercise can reduce your risk. However, the mechanisms responsible for this reduced risk are unknown. Several studies have linked exercise to improved memory, reduced amyloid beta plaques, and tau hyperphosphorylation in AD. **Background/Objectives**: By utilizing a novel rat model of AD, TgF344-AD, we evaluated the time course of behavioral shifts as well as the protective effect of exercise. **Methods**: TgF344-AD animals (61 total, 31 females and 30 males) were assessed every 3 months from 3 to 12 months of age and then assessments were increased to monthly until they reached 18 months of age. A progressive treadmill protocol was administered at 12 months of age and continued until 18 months. Pre-intervention and post-intervention data were analyzed. **Results**: Females had greater grip strength relative to body mass compared to males and exercise attenuated the age-related and AD-induced decline. Also, female AD-impaired memory was rescued with exercise, while males had no exercise-induced improvements. **Conclusions**: There is a sex difference present in the TgF344-AD rat model of Alzheimer’s disease and this should be studied further; in addition, sex differences across all models of AD and the human pathology need to be evaluated. Exercise neuroprotection, while more prominent in females, is an important factor in AD research, and further work to understand the mechanisms of neuroprotection is warranted.

## 1. Introduction

An estimated 416 million persons worldwide are living on the AD continuum (AD dementia, prodromal AD, and preclinical AD) [1]. The estimated global population of individuals 50 years of age and older is 1.9 billion people; thus, 22% of those 50+ are living on the AD continuum [1]. The number of individuals with AD dementia is estimated at 32 million globally and rising, with low- and middle-income countries expected to see the greatest increases by 2050 [2]. Diagnosis is typically initiated by family caregivers, and even with proactive caregivers, at the time of diagnosis, the disease has been present for nearly 20 years.

Exercise has been shown to prevent and slow the decay of white and gray matter in the brain—specifically in areas that are most affected by AD, such as the hippocampus (memory), prefrontal cortex (cognition), lateral temporal lobe (language processing), parietal lobe (cognition), and the corpus callosum (signal integration) [3,4,5,6,7]. The majority of AD research focuses on the hippocampus; this center for learning and memory is the earliest and often most severe area of atrophy in the AD brain. With natural aging beginning at around 50 years, the hippocampus undergoes a 1–2% decline in volume each year; for those with AD, their hippocampus will decline 3–5% in volume annually [8]. While more research is needed, it is clear that exercise can not only prevent this decline but that it can reverse it. In a study by Erickson et al. in 2011, the authors found a 2% increase in hippocampus volume following a 12-month exercise regimen in healthy older adults [6]. In a cross-sectional study, it was reported that increased physical activity was associated with reduced brain atrophy in AD patients [3]. As declines in hippocampus volume take extensive time and money to identify, impaired memory and cognition (additional hallmarks of the disease) are typically used to diagnose AD [9,10,11]. Indeed, exercise can effectively improve memory and cognition as well [4,12,13]. Despite this work, we are far from understanding the effects of exercise. What we do know, however, is that approximately one-third of AD cases worldwide are attributable to seven modifiable risk factors: diabetes, mid-life hypertension, mid-life obesity, physical inactivity, depression, smoking, and low educational attainment [14]. With as many as 70% of American older adults reporting no physical activity (directly impacting five of those seven reported risk factors for AD), physical activity proves to be an important factor to understand [15].

In addition to memory impairments, individuals with dementia are at increased risk of falling [16]. In older adults with mild-to-moderate AD, 6 months of exercise was enough to significantly reduce the risk of falling [17]. This improvement could be due to many factors, including enhanced balance and motor coordination. AD transgenic mice displayed motor coordination impairment at 6 (hSYN/hAPP mice) and 12 months (hSYN/hAPP and hSYN mice), so prolonged exercise may have long-lasting impacts on health and longevity [18].

Declining strength is indicative of an increased risk of AD, and there is a link between muscle strength, AD, and cognitive decline in older persons [19,20]. In a cross-sectional, case–control study conducted by Burns et al. [3], individuals with early-stage AD had reduced lean mass compared with controls. While it has been documented in several studies that AD and dementia are often correlated with reduced physical activity, lean mass remains independently associated with brain volume and cognition after controlling for physical activity [21]. In addition, the skeletal muscle of 3xTg-AD mice is more prone to oxidative and inflammatory events, which may directly contribute to brain function through brain–muscle crosstalk mechanisms [22].

While previous work demonstrates the positive effects of exercise on AD brain function and molecular composition, gaps in our knowledge remain. There remains a paucity of information regarding these responses in a model that more realistically mimics the slow progression of AD onset. The present study aims to evaluate the effect of aerobic exercise on body mass, spatial memory and learning, coordination, and muscular strength in male and female TgF344-AD rats. To further the field by effectively evaluating the connection between exercise and AD protection through biochemical and histopathological examination in future studies, the characterization of exercise-induced behavioral responses in the TgF344-AD rat model is vital.

TgF344-AD rats express the human amyloid beta precursor protein (APP) gene with the Swedish mutation and human presenilin 1 gene (PSEN1) with the Δ exon 9 mutation [23]. Both transgenes are driven by the mouse prion promoter [24]. This model is the first rat model to display amyloid B plaque deposits within the range of the human syndrome, tau proteins with isoforms that represent the 6 tau human isoforms, and consistent and extensive neuronal loss in the cortical and hippocampal regions beginning at 6 months of age [24]. When this model was compared to a mouse model containing the same genetic mutations, the rat model was found to be superior in the magnitude and nature of cerebral amyloidosis [25], and its tau proteome holds a close resemblance to the human proteome [26,27]. Lastly, rats’ operant behaviors are much more similar to humans, which is pivotal for studying AD. As this model becomes more widely utilized, it is important to evaluate the behavioral phenotype time course in addition to the pathological time course. The purpose of this project was to quantify behavioral changes in males and females with age and exercise intervention.

## 2. Materials and Methods

### 2.1. Experimental Animals

A breeding pair of TgF344-AD rats was obtained from Terrance Town at the University of Southern California. Male TgF344-AD rats were paired with two Fischer 344 female rats in breeding cages. TgF344-AD is a hemizygous strain developed through co-injection of two transgenes: (1) human amyloid-β precursor protein (AβPP) with the Swedish mutation and (2) human presenilin 1 (PSEN1) with a deletion of exon 9—both genes driven by the mouse prion promoter. These animals were bred and later housed two per cage in the Kansas State University (K-State) Comparative Medicine Group facility on a 12:12 light–dark cycle with food and water ad libitum. At 3 weeks of age, the animals were genotyped to identify those rats carrying the AD mutation. Roughly 50% of each litter will carry AD-related mutations. Based on the presence of AD, the animals were placed in either a wild-type control group (WT) or an Alzheimer’s disease group (AD). Additionally, the animals were assigned to either sedentary (SED) or exercise training (EX) groups. The number of animals per treatment group was as follows: female WT-SED (*n* = 12), female WT-EX (*n* = 5), female AD-SED (*n* = 8), female AD-EX (*n* = 6), male WT-SED (*n* = 6), male WT-EX (*n* = 8), male AD-SED (*n* = 8), and male AD-EX (*n* = 8). At 12 months of age, animals in the EX group began a treadmill training program five days per week. Behavioral data were collected at 3, 6, and 9 months of age and then monthly from 12 to 18 months.

### 2.2. Progressive Treadmill Training Protocol

At 12 months of age, following one week of acclimatization, animals in the exercise group began the progressive treadmill training protocol 5 days per week for 6 months. TgF344-AD animals display early cognitive deficits at 6 months of age, with pronounced learning impairment and significant neuronal loss at 15 months [24,28]. Animals in the sedentary group were restricted to cage activity with limited physical activity related to the behavioral testing, to which both SED and EX groups were identically exposed. Training was completed during the animal’s dark cycle and began at 15 meters/minute (m/m) for 15 min, progressing up to 24 m/m for 60 min. At 16 months of age, the intensity was decreased to 18 m/m, and the run time declined to 40 min. This intensity and duration were maintained for the remainder of the study. This exercise regimen is considered moderate intensity and has previously resulted in an exercise effect in animals of this strain and age [29]. Both TgF344-AD and wild-type controls were trained on the same schedule, and all training was completed during the animals’ dark cycle.

### 2.3. Behavioral Testing

#### 2.3.1. Grip Strength

Similar to human tests, rodent grip strength can be used as an indirect measure of total body strength. To assess the AD- and age-related changes in grip strength, each animal was removed from its cage by gripping the base of the tail between the thumb and forefinger. Then, the animal was gently lowered over the top of the rectangular grid attached to the grip strength meter (BioSeb, Havard Apparatus, Holliston, MA, USA) so that all 4 paws grip the grid. The animal was allowed to attach to the grid properly before being pulled away. The torso was kept horizontal as the rat was pulled back steadily until its grip was released. The maximal grip strength is achieved when the animal releases the bar. The maximal force is measured in grams and reported relative to body weight.

#### 2.3.2. Rotarod

Motor coordination is often reduced with age and AD. In addition, motor coordination impairments contribute to overall frailty seen with age and AD. As a measure of motor coordination, the rotarod test was conducted on all animals following the grip strength test. After sufficient acclimation with the rod (i.e., 60–120 s), animals from the same cage were placed in separate lanes on the rod set to rotate at 4 rpm. Utilizing a ramp Rotarod (Touchscreen RotaRod, Panlab, Harvard Apparatus, Holliston, MA, USA) protocol, the apparatus accelerated from 4 rpm to 40 rpm over a period of 120 s [27]. The trial began when acceleration started and ended when the animal fell from the rod. The procedure was repeated for a total of three trials separated by 15 min intervals. Fall time and speed at fall were recorded for each animal and trial.

#### 2.3.3. Morris Water Maze

Spatial learning and memory declines are commonly quantified with AD and age; in the present experiment, spatial learning and memory were assessed using the Morris Water Maze [30]. Briefly, the tank was filled with water to 1 inch above the platform. Nonfat dry milk was used to make the water opaque. Under a red light and at a water temperature of 25 °C, and following an acclimatization period, the animal was placed in the pool. The animal was allowed 60 s to find the hidden platform using the visual cues learned during the acclimatization period. The time-to-platform was recorded as the outcome measure, and shorter times are indicative of better memory of the platform’s location. The animal was directed to the platform if it was not able to find the platform within 60 s on their own, and the data were censored by recording these trials as a time-to-platform of 60 s. Upon completion of the trial, the animal was dried and returned to its cage.

### 2.4. AD Histopathology

Fixed (paraffin-embedded) brain tissue was sectioned (5–10 μm) with a microtome (Leica RM2235, Wetzlar, Germany). Brain sections were processed and stained with H&E, Luxol-Fast Blue, and Thioflavin-S. The whole-tissue micrographs of the sections were obtained using a slide scanner (PathScan Enabler IV) coupled to a transmitted light microscope (Leica DM1000). Vibratome free-floating sections (40 µm) were processed and immunostained with anti-beta amyloid (6E10, Covance, 1:400, Princeton, NJ, USA) and anti-Tau (PHF-1, Albert Einstein College of Medicine, Bronx, NY, USA, 1:1000) as previously described [31]. The monoclonal mouse 6E10 antibody is reactive to the amino-acid residues 1–16 and the epitope lies within amino acids 3–8 of beta amyloid. For single labeling, all sections were washed with 0.1 M Tris-buffered saline (TBS), pH 7.4, and then pretreated with 3% hydrogen peroxide in 10% methanol to block endogenous peroxidase activity. The sections were subsequently washed in TBS with 0.1% Triton X-100 (TBS-A) and then blocked for thirty minutes in TBS-A with 3% bovine serum albumin (TBS-B). The sections were further incubated overnight at room temperature with the primary antibodies. Following two washes with TBS-A and a wash in TBS-B, the sections were incubated in anti-rabbit or mouse biotinylated anti-IgG (1 h) and then in avidin biotin complex (1 h) (ABC, Elite Immunoperoxidase, Vector Laboratories, Burlingame, CA, USA). The primary antibody was visualized using a brown DAB substrate (Vector Laboratories). To visualize beta-amyloid staining, the sections were pretreated for 5 min in 95% formic acid. PHF-1 was a generous gift from Dr. Peter Davies (Albert Einstein College of Medicine, Bronx, NY, USA) and was used at 1:1000. Bright field images of were captured at 20× magnification and plaque density was estimated using ImageJ Version 1.52 according to a modified method [31]. The microscope slides were visualized under 20× on an Olympus light microscope. Using MagnaFire, a piece of digital microscopy software, three images of different parts of the hippocampus from each section were taken. The images were then analyzed using Image J, a scientific image analyzer.

In Image J, each image was converted to a 1280 × 1024 pixel, 8-bit type. The highlighting threshold was adjusted as needed to highlight the plaques in each image. The percent area covered with plaques within each image was then measured. An average percent area covered with plaques was calculated for all three images of each section and then an average percent area covered with plaques in each animal was calculated. A final average of the percent area covered with plaques was taken across all sedentary animals and was compared to that of the exercised animals.

### 2.5. Statistical Analysis

Bayesian multivariate repeated measures linear regression analysis was utilized to probe for differences. Multivariate analysis of all four outcomes simultaneously reduces the likelihood of producing errors due to multiple tests across multiple groups (i.e., it controls for Type 1 error). Bayesian analysis was necessary to perform a multivariate repeated measures analysis while fitting grip strength, rotarod, and maze data using a lognormal distribution. Variables reflecting a real difference were identified as those with a 95% credible interval that did not contain zero. The brms package developed in the *R* platform Version 1.52was used with this data set. For the analysis of amyloid and tau pathology, GraphPad Prism Version 10.4.0 was used to perform *t*-tests between AD-SED and AD-EX.

## 3. Results

### 3.1. General Characteristics

The experiment included 62 animals, animals per experimental group: WT males (*n* = 14), WT females (*n* = 17), AD males (*n* = 17), and AD females (*n* = 14). Animals tolerated the exercise protocol well with only one non-compliant runner excluded (AD male). An animal is returned to their home cage during an exercise session if they sit along the back wall for more than 3 s at a time more than 5 times in a session. If an animal had not completed three consecutive exercise sessions, they were determined to be non-compliant and removed from the study.

### 3.2. Body Mass Gains Are Blunted with Advanced Age and EX

**Figure 1:**
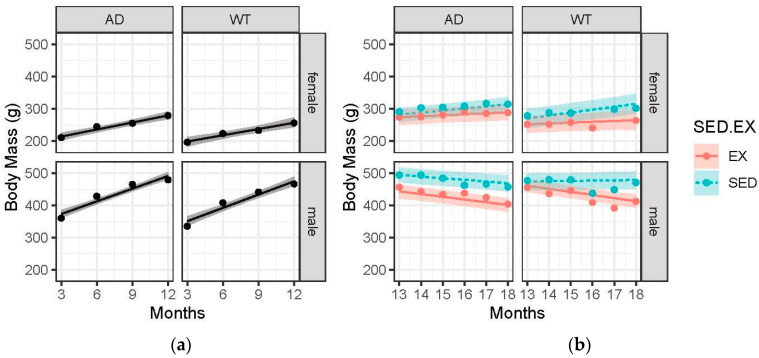
Body mass (grams) over time across the 4 treatment groups: female-AD, female-WT, male-AD, male-WT. Circle data points represent the group mean and error ribbons are +/− 1 SE of the mean. (**a**) Pre-intervention data collected at 3, 6, 9, and 12 months. (**b**) Post-intervention data collected monthly from 13 to 18 months. Experimental groups were further divided into two interventions: exercise (EX) and sedentary (SED).

As expected, the male rats weighed more than the female rats at every time point, Figure 1. Pre-intervention, AD rats (both males and females) had a greater mass than the WT controls (AD male: 492 grams (g) v WT male: 475 g and AD female: 280 g v WT female: 257 g at 12 months). However, post-intervention, there was no appreciable difference between AD and WT (the difference was 4 g with an estimated error of 4 g). Male rats gained at a greater rate than the female rats pre-intervention and lost weight post-intervention, while females continued to gain (female: 6.9 grams/month (g/m) pre- and 5.4 g/m post- and male: 13.5 g/m pre- and −5.6 g/m post-). Interestingly, the AD sedentary females gained weight from 13 to 18 months (7.9 g) while the AD male sedentary animals lost weight (−2.2 g).

### 3.3. Females Had Greater Grip Strength Relative to Body Mass Compared to Males, and Exercise Trended Towards Attenuating the Age-Related and AD-Induced Decline

**Figure 2:**
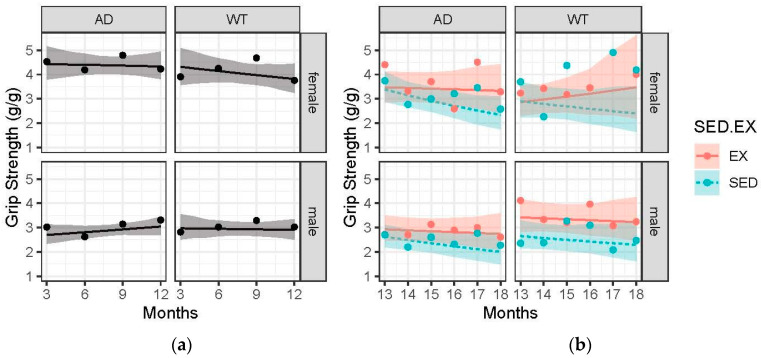
Grip strength (grams/body mass in grams) over time across the 4 treatment groups: female-AD, female-WT, male-AD, male-WT. Circle data points represent the group mean, and error ribbons are +/− 1 SE of the mean. (**a**) Pre-intervention data collected at 3, 6, 9, and 12 months. (**b**) Post-intervention data collected monthly from 13 to 18 months. Experimental groups were further divided into two interventions: exercise (EX) and sedentary (SED).

Pre-intervention, females had greater grip strength in both AD (4.43 to 4.34 g/g) and WT (4.32 to 3.82 g/g) compared to male AD (2.70 to 3.04 g/g) and WT (2.97 to 2.91 g/g), Figure 2a. The change was assessed based on the best-fitting slope of the change in grip strength per month on the log-transformed g/g scale. Post-intervention, exercise trended towards attenuated declines in strength, with sedentary animals declining −0.050 per month compared to a near-absent change in exercised animals (+0.001). While the 95% CI did contain zero (0.03, 0.18), this result suggests a maintenance effect of exercise across sex and AD presence with age. In addition, AD animals experienced a greater decline in strength compared to their WT controls (−0.038 vs. 0.011 log-transformed g/g per month), but again, this was not statistically significant with a 95% CI, −0.08–0.07. Lastly, while both males and females experienced exercise-induced improvements, females’ improvements superseded those of the males. Female AD exercisers’ change in strength between 13 and 18 months of age was 8.5 times greater (non-significant) than the female AD sedentary group (−0.009 vs. −0.075 log-transformed g/g per month scale), Figure 2b, while that of the male AD exercisers was only 4 times greater (non-significant) than the male AD sedentary group (−0.013 vs. −0.054). As displayed by the greater slope differences between EX and SED in AD animals, Figure 2b.

### 3.4. Females (WT and AD) Performed Better on the Rotarod Compared to Males. Performance Pre-Intervention Declined with Age Across All Groups and Largely Reached an Asymptote in Post-Intervention Analysis

**Figure 3:**
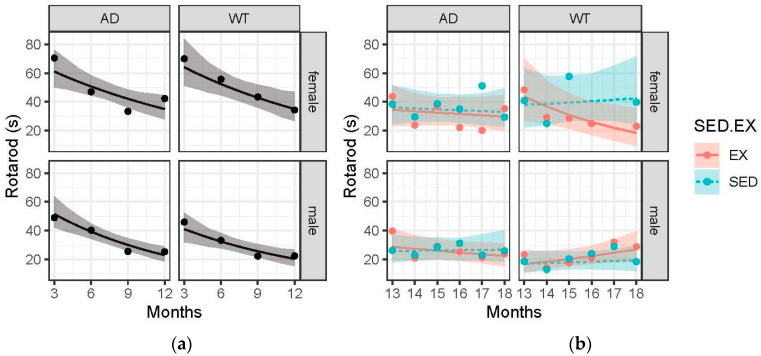
Rotarod (seconds) over time across the 4 treatment groups: female-AD, female-WT, male-AD, and male-WT. Circle data points represent the group mean and error ribbons are +/− 1 SE of the mean. (**a**) Pre-intervention data collected at 3, 6, 9, and 12 months. (**b**) Post-intervention data collected monthly from 13 to 18 months. Experimental groups were further divided into two interventions: exercise (EX) and sedentary (SED).

With a greater time-to-fall indicative of greater motor coordination, we expected increased times to result from the exercise intervention. The presented results indicate no effect of AD pathology or exercise on motor coordination as measured with the Rotarod (Figure 3). This negative result is likely due to the negligible sensitivity to detect smaller effects.

### 3.5. Memory in Female AD Animals Is More Likely to Be Positively Impacted by Exercise Compared to Male AD Animals

**Figure 4:**
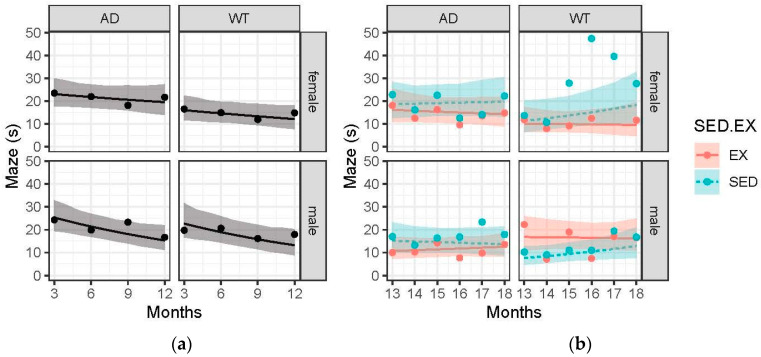
Maze (seconds) over time across the 4 treatment groups: female-AD, female-WT, male-AD, male-WT. Circle data points represent the group mean, and error ribbons are +/− 1 SE of the mean. (**a**) Pre-intervention data collected at 3, 6, 9, and 12 months. (**b**) Post-intervention data collected monthly from 13 to 18 months. Experimental groups were further divided into two interventions: exercise (EX) and sedentary (SED).

In Figure 4a, all groups improved their maze performance as measured by latency time to find the hidden platform in the water maze from 3 months until 12 months of age. Female AD animals’ average latency time was higher than the female WT controls at 3, 6, 9, and 12 months (3 months: female AD 23.0 s vs. female WT 16.1 s, 6 months: female AD 21.8 s vs. female WT 14.6 s, 9 months: female AD 20.7 s vs. female WT 13.3 s, and 12 months: female AD 19.6 s vs. female WT 12.1 s). Males trended towards a greater rate of improvement (−0.058 vs. −0.025 log scale) compared to females from 3 to 12 months of age. The post-intervention data (Figure 4b) reveal a possible effect of exercise in females only. The female exercise group trended towards improved latency time from 13 to 18 months (−0.02 log scale s/m) while the female sedentary group maintained or waned their latency time. Males (both AD and WT) did not show a real effect of exercise on memory.

### 3.6. Exercise Significantly Decreased AD Pathology

**Figure 5:**
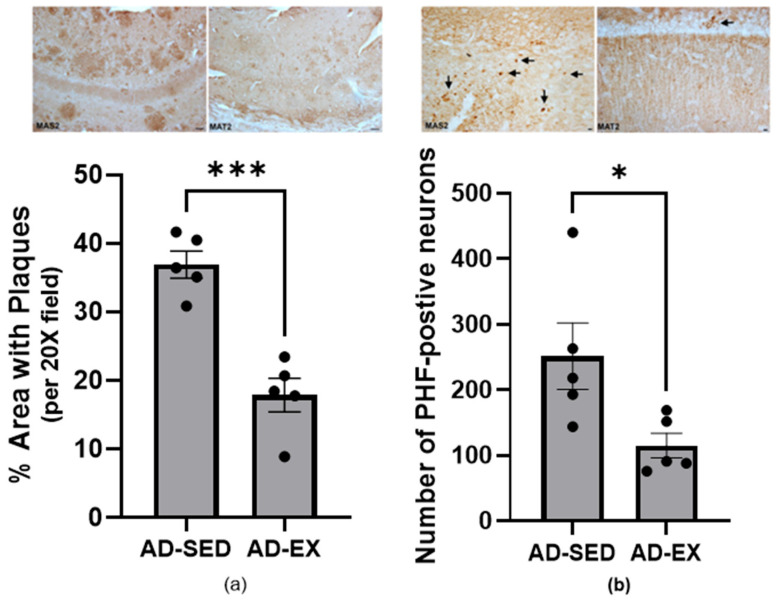
Hippocampus proper brain sections from 18-month-old TgF344-AD rats under sedentary (SED) conditions or following 6-month treadmill training (EX). Each group contained 5 animals (3 males and 2 females); the points are individual animal values, and bars are group means with SEMs. (**a**) Amyloid plaques were stained with 6E10 and quantified as % area with plaques. The top panels show staining in the hippocampus proper under sedentary conditions (left) or exercise (right), both from a male sample. Exercise resulted in a significant decline in amyloid plaques (*** *p* < 0.001). (**b**) The top panel shows pathologically phosphorylated tau at S396/404 (PHF) positive neurons in the hippocampus (CA1) of male sedentary (left) and exercise (right) rats. The proportion of PFT-positive neurons significantly decreased following exercise compared to the sedentary AD animals (* *p* < 0.05).

Utilizing a smaller subset of animals (five AD-SED and five AD-EX, each group with three males and two females), we determined that exercise significantly decreased the % area covered with amyloid plaques (Figure 5a) and the number of PHF-positive neurons (indicative of tau tangles) in the hippocampus proper (Figure 5b). This confirms exercise protection in 18-month-old TgF344-AD rats following a 6-month treadmill training intervention.

## 4. Discussion

Interestingly, the body mass shifts were distinctly different in males and females. Our post-intervention data show that female AD animals (both sedentary and exercise) showed increased body mass from 13 to 18 months, while the male AD animals (both sedentary and exercise) showed decreased body mass. Previous work in the TgF344-AD model would lead us to believe that the female gains would be largely due to increased fat mass [25,26]; however, to date, no work has been published to explain the sex differences seen in the TgF344-AD rat. If the increased body mass in females was the result of increased fat mass, it begs the question: What if increased fat mass was induced with a high-fat diet? And what effects would that have on AD phenotype? While these questions have not been addressed in TgF344-AD animals, they have been addressed in mouse models of AD. High-fat mass induced with a high-fat diet has been associated with increased amyloid-beta and tau pathologies as well as declines in cognition; however, no effect of sex was identified [27,28,32]. While the sex differences in body mass changes are evident in this project, more work is needed to understand if the shifts are due to fat mass changes.

This is the first report of skeletal muscle strength declines in the TgF344-AD rat. AD sedentary animals (both males and females) had declines in strength, which were slightly greater than the age-related declines seen in WT sedentary animals. Mouse models of AD have confirmed that declines in strength are found in AD animals, both males and females [33,34]. In addition, when skeletal muscle function and mass are compromised with induced muscle atrophy via limb casting, the cognitive decline in 5xFAD mice was significantly accelerated [35]. Overexpression of myostatin documented in APP/PS1 mice could be the cause of reduced skeletal muscle mass and function [36]. In fact, when myostatin is knocked down in APP/PS1 mice, the AD animals showed an increase in grip strength, muscle mass and cognition [36]. While skeletal muscle loss and strength loss are closely associated with increased risk of developing AD and correlated with AD severity, relationships are still correlative, and more work is needed to determine the role of skeletal muscle in brain health [20,37,38]. Lastly, it is important to note that all exercise groups improved their strength compared to their sedentary counterparts. This is likely due to the preservation of skeletal muscle mass and function.

Females outperformed males in motor coordination (Rotarod) across all groups. This difference is attributable to the decreased body weight, as several studies have indicated a significant role of mass [33,34]. While our data do not suggest AD animals outperformed WT, previous research in mouse models has found that to be the case [23,39,40,41]. Lastly, the pre-intervention declines suggest a negative learning effect, and thus, repeated rotarod assessment is not recommended because repetition might habituate rats to falling. AD patients have documented increased frailty due to loss of skeletal muscle mass and loss of motor coordination. Specifically, gait disorders, loss of balance, and postural impairments are all affected in AD and impact motor coordination [42]. Understanding the mechanism of motor coordination impairments, alongside skeletal muscle atrophy, will go a long way in improving the quality of life of AD patients and their caregivers.

Memory changes were as expected, with the AD groups showing impaired maze performance compared to the WT groups. In addition, the AD exercise groups showed improved memory compared to the AD sedentary groups, which promotes the use of exercise as an AD treatment. One study to date found that exercise in the TgF344-AD model did not have an effect on hippocampus-dependent recognition memory at 12 months but did improve fear avoidance and anxiety-like behaviors [43]. This outcome is likely due to the early age of assessment. While our data did not reveal a strong exercise effect in male AD animals, our female group displayed a protective effect at 18 months of age. Indeed, it is very well documented among other AD animal models that exercise protects against memory impairment [44,45,46,47]. The mechanisms of exercise-induced neuroprotection include increased neurogenesis (via BDNF), reduced neuroinflammation, increased cerebral blood flow, and improved mitochondrial function [48]. In addition, female mice and rats often show a greater response to exercise interventions than males [49]. However, this finding is independent of sex hormones [49]. What does seem to be sex hormone-dependent is hippocampal memory [50], such as spatial memory or recognition memory, which may have contributed to our females performing better on memory tasks compared to males. Continued research is needed to understand the mechanisms of exercise-induced neuroprotection and the role of sex.

The current experiment utilized an aerobic exercise intervention. Aerobic exercise has been closely associated with improved cognition in middle-aged and older healthy adults [51] and in older, mildly cognitively impaired adults [52]. Resistance exercise has also been shown to improve cognition in animal models of AD [53,54]; however, the results are mixed in MCI or AD patients [55,56,57]. Flexibility and balance exercises stress the neuromuscular demands in exercise and can lead to cognitive improvement through the complexity of movement. Tai Chi has gained a lot of interest in its effects on brain health. In healthy older adults, Tai Chi training with its low-impact, slow, intentional movements showed greater cognition improvements compared to brisk walking [58].

## 5. Conclusions

This experiment is the first to document skeletal muscle strength declines in the TgF344-AD rat and reveal prominent sex differences in body mass, motor coordination, and memory. While female AD rats gained body mass with age, males lost body mass, which suggests a sex-specific age-related metabolic change. Exercise preserved muscle strength and improved memory performance in females, emphasizing its role as a non-pharmacological intervention in AD. Continued research is needed to clarify the sex-specific mechanism and optimize exercise modalities for AD prevention and treatment.

## 6. Limitations

In the present study, a transgenic animal model of a human disease was utilized to gain further insight into the time course of the disease. The purpose was to inform future research with this animal model. However, we believe that the results could, to some extent, be translated to the human disease. This translation should consider that exercise was implemented at 12 months of age to evaluate its effectiveness in treating the AD pathology. However, exercise throughout the lifespan has a positive impact on brain health [59]. The human brain reaches full maturity in one’s mid-20s and begins to shrink in one’s 40s and 50s (roughly 5% every 10 years) until 70 years of age, at which the rate of decline increases even further [60]. This experiment implemented an exercise intervention following full brain maturity and the onset of AD pathology. Further experimentation should be conducted to understand the exercise-induced neuroprotection during earlier ages. Brain plasticity is at its peak during adolescent years, making this an important life phase to study; indeed, some studies suggest that early life exercise performed during peak brain plasticity years can provide protection into adulthood even after inactivity in mid- and late life [61].

## Data Availability

The data presented in this study are available from the corresponding author upon request.
